# Une fracture pertrochanterienne révélant un myélome multiple: quelle prise en charge?

**DOI:** 10.11604/pamj.2017.27.155.12069

**Published:** 2017-06-30

**Authors:** Hicham Bousbaa, Mourad Bennani, Naoufal Elghoul, Mohammed Ouahidi, Jamal Louaste, Laarbi Amhaji

**Affiliations:** 1 Service d’Orthopédie-Traumatologie, Hôpital Militaire Moulay Ismail, BP S 15, 50000 Meknès, Maroc

**Keywords:** Myélome multiple, fracture per trochantérienne, clou gamma, Multiple myeloma, pertrochanterian fracture, gamma nail

## Abstract

L’atteinte squelettique est la complication clinique majeure au cours des myélomes multiples avec une part non négligeable pour les fractures pathologiques. Les fractures du fémur proximal sont très fréquentes au cours de l’évolution du myélome multiple et elles compromettent sérieusement la survie ainsi que la qualité de vie du patient cancéreux. Un traitement chirurgical précoce permet l’amélioration de la mortalité et la morbidité. L’ostéosynthèse par clou cervicomédullaire verrouillé de type clou gamma constitue un moyen stable, efficace et durable qui permet la levée précoce et une amélioration de la survie du malade. Cependant, une survie prolongée impose une surveillance régulière de l’ostéosynthèse pour dépister et traiter une faillite de matériel.Il faut en dernier lieu ne pas perdre de vue que le regain de l’autonomie après une fracture pertrochantérienne hypothèque même le contrôle du myélome par la possibilité ou non de réaliser l’autogreffe de la moelle osseuse.

## Abstract (en)

Skeletal involvement is the major clinical complication of multiple myelomas, resulting in pathological fractures. Proximal femoral fractures are very frequent during multiple myeloma evolution and they seriously compromise survival and quality of life of patients with cancer. Early surgical treatment allows mortality and morbidity improvement. Cervicomedullary Gamma locking nail osteosynthesis allows efficient, sustainable and stable fixation associated with early patient mobilization and survival improvement. However, prolonged survival imposes regular monitoring of the osteosynthesis in order to detect and to treat equipment failure. We should not overlook the fact that the recovery of independence after pertrochanterian fracture also influences myeloma control through the possibility to perform bone marrow autograft or not.

## Introduction

L’atteinte squelettique est la complication clinique majeure au cours des myélomes multiples avec une part non négligeable pour les fractures pathologiques. Les fractures du fémur proximal sont très fréquentes au cours de l’évolution du myélome multiple et elles compromettent sérieusement la survie ainsi que la qualité de vie du patient cancéreux. Un traitement chirurgical précoce permet l’amélioration de la mortalité et la morbidité. Nous rapportons le cas d’une patiente, atteinte d’une fracture pertrochantérienne qui a révélé un myélome multiple et qui a été traitée par une ostéosynthèse par clou gamma statique.

## Patient et observation

**Figure 1:**
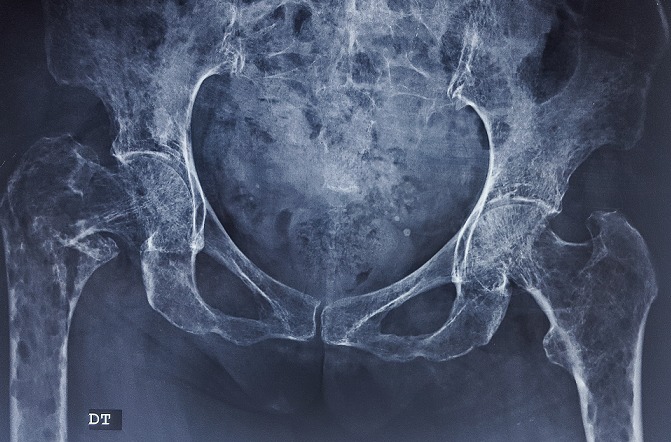
Radiographie de la hanche de face: fracture pertrochantérienne du fémur droit avec un large fragment du petit trochanter; multiples images lacunaires disséminées dans les deux fémurs et les os du bassin

**Figure 2:**
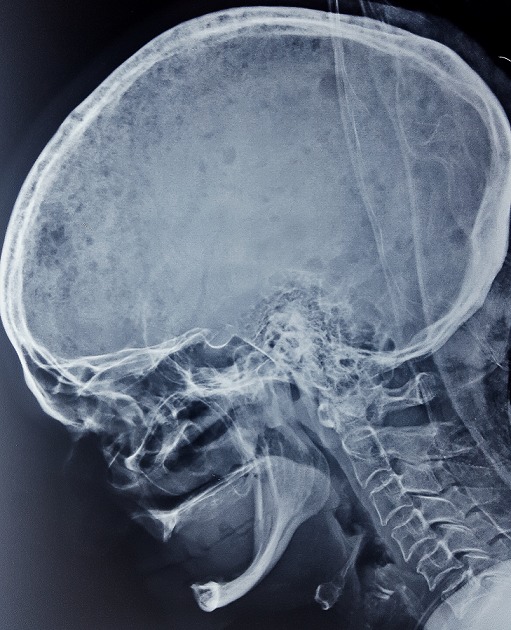
Radiographie du crâne: lésions lacunaires en « emporte-pièces » caractéristiques du myélome multiple

**Figure 3:**
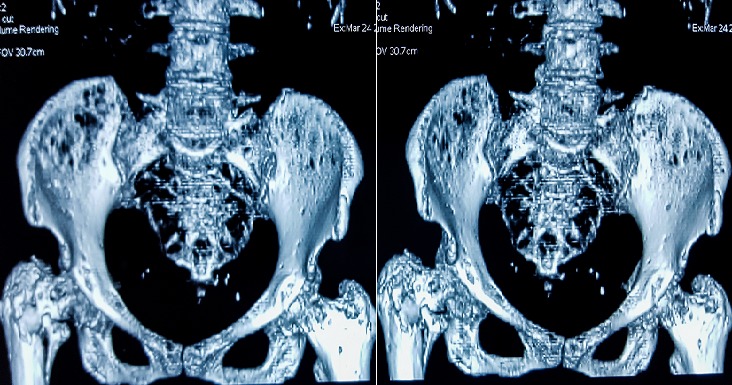
TDM du bassin avec reconstruction 3D permettant une meilleur caractérisation de la fracture et des images lacunaires osseuses associées

**Figure 4:**
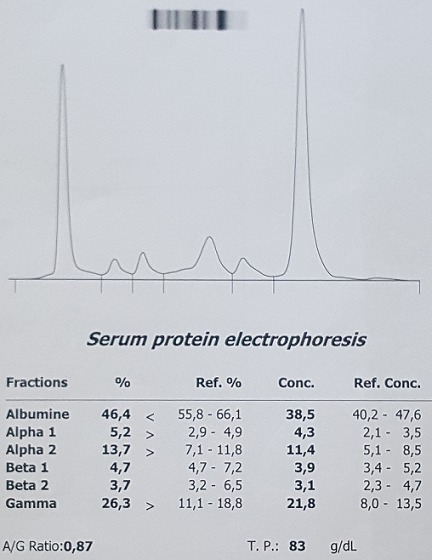
Électrophorèse des protéines sériques: pic étroit migrant dans la zone des gammaglobulines

**Figure 5:**
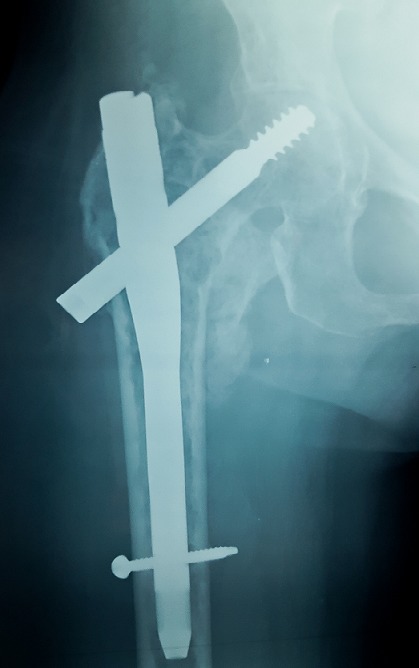
Radiographie de la hanche droite de face montrant une bonne consolidation osseuse avec une ostéosynthèse stable après 6 mois de recul

Il s’agit d’une femme âgée de 58 ans, sans antécédents médico-chirurgicaux notables, qui avait consulté aux urgences pour une cruralgie aigue avec impotence fonctionnelle totale du membre inférieur sans notion de traumatisme. L’examen clinique à l’admission a retrouvé une patiente consciente et stable sur le plan hémodynamique et respiratoire, avec un membre inferieur droit raccourci, déformé en adduction et en rotation externe. Une radiographie de la hanche de face a été réalisée (Figure 1) et a objectivé la présence d’une fracture pertrochantérienne du fémur droit avec un large fragment du petit trochanter; en outre elle a montré de multiples images lacunaires disséminées dans les deux fémurs et les os du bassin. Une radiographie du crâne (Figure 2) a montré des lésions lacunaires en « emporte-pièces ». Une tomodensitométrie (TDM) du rachis et du bassin (Figure 3) a permis une classification précise de la fracture en type IV selon la classification de Jensen-Michaëlsen [1] et 31A2.1 selon la classification de l’AO [2]. La TDM a aussi objectivé l’aspect disséminé des lacunes sur l’ensemble du squelette axial. Le mécanisme non traumatique de la fracture ainsi que les images radiographiques nous ont fait retenir le diagnostic d’une fracture pertrochantérienne instable sur os pathologique. Le myélome multiple a été confirmé sur la présence d’un pic étroit migrant dans la zone des gammaglobulines à l’électrophorèse des protéines sériques (Figure 4), d’une bande monoclonale IgG kappa à l’immunofixation et d’une infiltration plasmocytaire médullaire à 15% au myélogramme. La patiente a été traité chirurgicalement par une ostéosynthèse intramédullaire à foyer fermé de type clou gamma verrouillé avec un montage statique. Les suites post-opératoires ont été normales. La mobilisation précoce à été autorisée, une période de décharge de 4 semaines a été observée sous couvert d’une héparinothérapie de bas poids moléculaire, et un traitement par bisphosphonates a été débuté (acide zoledronique à raison d’une perfusion par mois). La patiente est suivie en coordination avec le service d’hématologie clinique où elle a été mise sous un protocole de chimiothérapie d’induction (bortézomib, thalidomide et dexaméthasone). Après 6 mois, la patiente a été revue en consultation en bon état général et autonome. L’évaluation clinique par la cotation de Postel et Merle d’Aubigné (PMA) a retrouvé un score de 16. La radiographie de contrôle de la hanche droite a montré une ostéosynthèse stable et une fracture consolidée (Figure 5). L’autonomie de la patiente a été décisive pour la possibilité de réaliser une autogreffe de la moelle osseuse après un traitement d’induction et donc pour un contrôle de la maladie myélomateuse.

## Discussion

Le myélome multiple est une hémopathie maligne caractérisée par la prolifération d’un clone de plasmocyte envahissant la moelle hématopoïétique. Il atteint le plus souvent le sujet âgé, et les manifestations osseuses (douleurs, fractures pathologiques) dominent fréquemment le tableau clinique. Le diagnostic est facile, sur l’association d’une plasmocytose médullaire excessive et d’une immunoglobuline monoclonale sérique et/ou urinaire [3]. L’atteinte squelettique est la complication clinique majeure au cours des myélomes multiples. En effet, 70% des patients présentent des lésions d’ostéolyse avec ou sans ostéoporose [4]. Les fractures pathologiques touchent 40% des patients atteints d’un myélome compromettant la survie et la qualité de vie [5]. Le fémur est l’os long le plus affecté par les métastases osseuses, surtout par les cancers dit ostéophiles: sein, rein, prostate, poumon, colon, thyroïde; le 1/3 en localisation proximale [6]. Les fractures causées par un myélome peuvent être gérées comme celle dues à un carcinome, et leur comportement biologique ainsi que leurs implications mécaniques sont similaires aux fractures métastatiques [7]. A cause de son rôle majeur dans le support du poids du corps, la fracture pathologique du fémur détériore sérieusement la qualité de vie du patient cancéreux. Le problème d’un diagnostic étiologique se pose en premier et une investigation rapide et ciblée nous a permis de confirmer le diagnostic de myélome multiple. La TDM osseuse possède une meilleure sensibilité et spécificité pour bien caractériser les lésions ostéolytiques et, grâce à la reconstruction 3D, elle permet une étude précise de la fracture ; aussi, elle allie un court temps d’exposition à une large exploration respectant ainsi le confort du malade [8].

Différentes options chirurgicales sont disponibles pour permettre une fixation solide et durable des fractures pathologiques du massif trochantérien du fémur. Le curetage à ciel ouvert avec une fixation par lame plaque ou DHS en utilisant le ciment acrylique sont indiqués pour les lésions métastatiques incluant moins que la moitié de l’épiphyse ou la métaphyse, mais ils ont montré un taux inacceptable de faillite du matériel une fois les métastases progressent [7, 9]. L’enclouage cervicomédullaire verrouillé est actuellement indiqué pour traiter des patients avec de multiples métastases osseuses quand la région trochantérienne et la diaphyse fémorale sont principalement concernées [10]. C’est une technique peu invasive, et qui permet de prévenir les fractures causées par une extension tumorale ou une autre localisation dans le même os [6]. De plus, L’ostéosynthèse par clou centromédullaire verrouillé assure une mobilité post-opératoire avec déambulation précoce et une amélioration de la qualité de vie au même titre que la survie. En effet, si la survie moyenne semble chuter d’une moyenne de 57 mois à 18 mois après une fracture pathologique au cours d’un myélome, elle augment après la chirurgie à 47 mois [5]. Cependant, le taux de survie post-opératoire est considéré comme le principal facteur de risque de faillite du clou cervicomédullaire, les études ayant montré que quand les patients survivent plus que 3 ans après l’opération, ce risque augmente [11]; ce qui incite à considérer la résection et la reconstruction par prothèse pour les patients en bon état général avec un myélome de bon pronostic. Le complément thérapeutique par bisphosphonates est incontournable pour prévenir et traiter l’atteinte osseuse au cours du myélome multiple, que ce soit l’acide clodronique par voie orale ou l’acide zoledronique ou pamidronique par voie intraveineuse [12, 13]. L’American Society for Clinical Oncology (ASCO) a fixé en 2007 les modalités de ce traitement, avec une préférence pour les bisphosphonates en intra-veineux et une durée minimale de traitement de 2 ans à reconsidérer selon la réponse hématologique à l’auto-greffe [14]. Les résultats anatomiques et fonctionnels chez notre patiente, obtenus par une ostéosynthèse stable, efficace et durable, se sont traduits par un regain de l’autonomie, sans laquelle une autogreffe de la moelle osseuse ne peut être envisagée (séjour en chambre stérile).

## Conclusion

Le cas de notre patiente vient nous rappeler que la prise en charge d’une fracture pathologique du massif trochantérien commence dès sa suspicion, passant par un diagnostic positif précis et un diagnostic étiologique rapide, qui aboutissent à leur tour à un traitement adapté visant la levée précoce et le regain de l’autonomie, dans le cadre d’une surveillance multidisciplinaire. La TDM permet désormais d’allier le confort du malade, une exploration exhaustive des lésions osseuses dues au myélome multiple, et un soulagement de l’indication opératoire par une étude et une classification précises de la fracture. L’ostéosynthèse par clou cervicomédullaire verrouillé de type clou gamma constitue un moyen stable, efficace et durable qui permet la levée précoce et une amélioration de la survie du malade. Cependant, une survie prolongée impose une surveillance régulière de l’ostéosynthèse pour dépister et traiter une faillite de matériel. Il faut en dernier lieu ne pas perdre de vue que le regain de l’autonomie après une fracture pertrochantérienne hypothèque même le contrôle du myélome par la possibilité ou non de réaliser l’autogreffe de la moelle osseuse.

## Conflits d’intérêts

Les auteurs ne déclarent aucun conflit d'intérêt.
